# CT radiomics-based machine learning model for differentiating between enchondroma and low-grade chondrosarcoma

**DOI:** 10.1097/MD.0000000000039311

**Published:** 2024-08-16

**Authors:** Mustafa Yildirim, Hanefi Yildirim

**Affiliations:** a Firat University School of Medicine Department of Radiology, Elaziğ, Turkey.

**Keywords:** computed tomography, enchondroma, low-grade chondrosarcoma, machine learning, radiomics analysis

## Abstract

It may be difficult to distinguish between enchondroma and low-grade malignant cartilage tumors (grade 1) radiologically. This study aimed to construct machine learning models using 3D computed tomography (CT)-based radiomics analysis to differentiate low-grade chondrosarcoma from enchondroma. A total of 30 patients with enchondroma and 26 with chondrosarcoma were included in this retrospective study. Tumor volume segmentation was manually performed by 2 musculoskeletal radiologists. In total, 107 radiomic features were obtained for each patient. The intraclass correlation coefficient was used to assess interobserver reliability and estimate the absolute agreement between the 2 radiologists. Algorithm-based information gain was used as a feature reduction method, and the 5 most important features were detected. For classification, 7 machine learning models were utilized. Classification was carried out using either all features or 5 features. There was good to excellent agreement between the 2 radiologists for the 107 features of each patient. Therefore, a dataset containing 107 features was used for machine learning classification. When assessed based on area under curve (AUC) values, classification using all features revealed that naive Bayes was the best model (AUC = 0.950), while classification using 5 features revealed that random forest was the best model for differentiating chondrosarcoma from enchondroma (AUC = 0.967). In conclusion, machine learning models using CT-based radiomics analysis can be used to differentiate between low-grade chondrosarcoma and enchondroma.

## 1. Introduction

Enchondroma and chondrosarcoma are the most frequent benign and malignant cartilage forming bone tumors, respectively. Chondrosarcomas account for 20 to 27% of primary malignant bone sarcomas, while enchondromas account for 15.6% of benign bone tumors.^[1,2]^ Chondrosarcoma is classified as grade I, II, or III.^[3]^ Grade I chondrosarcomas show a good prognosis and rarely metastasize.^[4]^ According to current tumor terminology, it is appropriate to refer to low-grade tumors that originate from the long and short tubular bones of the extremities as “atypical cartilaginous tumors” or low-grade central chondrosarcomas.^[5]^ Surgical resection is the primary treatment method for chondrosarcoma. Enchondroma is a benign bone lesion; thus, a wait-and-see approach is acceptable.^[6]^

Cartilaginous tumors are often detected incidentally on radiological imaging.^[7]^ These tumors are easily identified on radiographs. High-grade chondrosarcomas (grades II–III) have distinct malignant features and therefore can be easily distinguished from enchondromas radiologically. However, it may be difficult to distinguish between enchondroma and low-grade malignant cartilage tumors (grade 1).^[8]^ For this reason, a combination of radiological, clinical, and histopathological findings is used for differential diagnosis.^[9]^ However, there are significant histological challenges in differentiating low-grade chondrosarcomas from enchondromas. In addition, numerous investigations have revealed inadequacies in identifying tumor morphology from a single tumor biopsy due to intratumor heterogeneity.^[9]^ Yet another challenge is that a bone biopsy is a difficult procedure for the patient to undergo. Therefore, many studies have been conducted to radiologically differentiate chondrosarcoma from enchondroma. Texture analysis, radiomics applications, and machine learning applications have been used for the diagnosis.^[10–12]^ Lisson et al used magnetic resonance imaging (MRI)-based texture analysis to differentiate low-grade chondrosarcoma from enchondroma.^[10]^ Erdem et al used MRI-based radiomics and machine learning classification for the differentiation of chondrosarcoma from enchondroma.^[11]^ Yoon et al used single photon emission computed tomography (SPECT/CT) radiomics analysis to distinguish between low-grade chondrosarcoma and enchondroma.^[12]^ Deng et al used MRI and CT-based texture analysis for grading cartilaginous bone tumors.^[13]^

To our knowledge, none of the studies reported to date have used CT-based radiomics analysis to distinguish between enchondroma and low-grade chondrosarcoma. The current study aimed to create machine learning models that can distinguish between low-grade chondrosarcoma/atypical cartilaginous tumors and enchondroma based on radiomic features extracted from CT images and compare the diagnostic value of machine learning models.

## 2. Materials and methods

**Figure 1.**
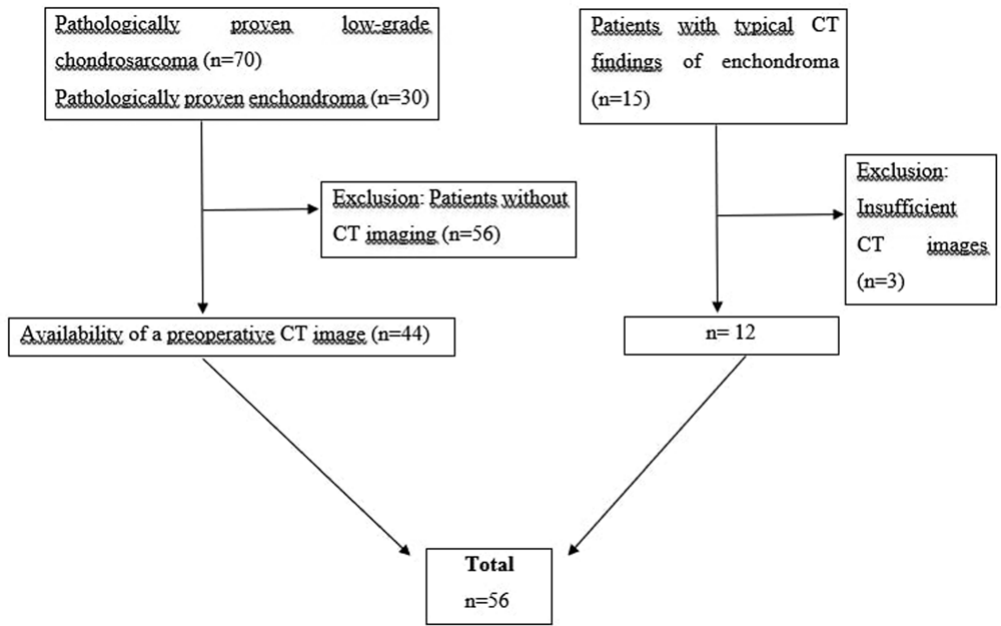
The flowchart showing patient selection.

**Figure 2.**
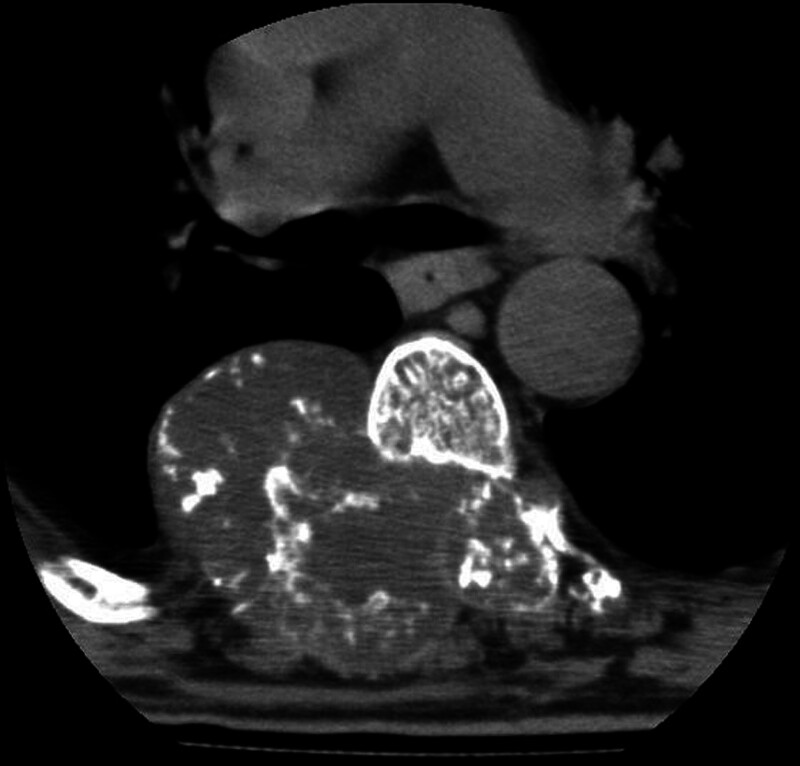
The axial CT image shows a soft tissue mass with a calcified matrix in the paraspinal location. Histopathological diagnosis was low-grade chondrosarcoma. CT = computed tomography.

**Figure 3.**
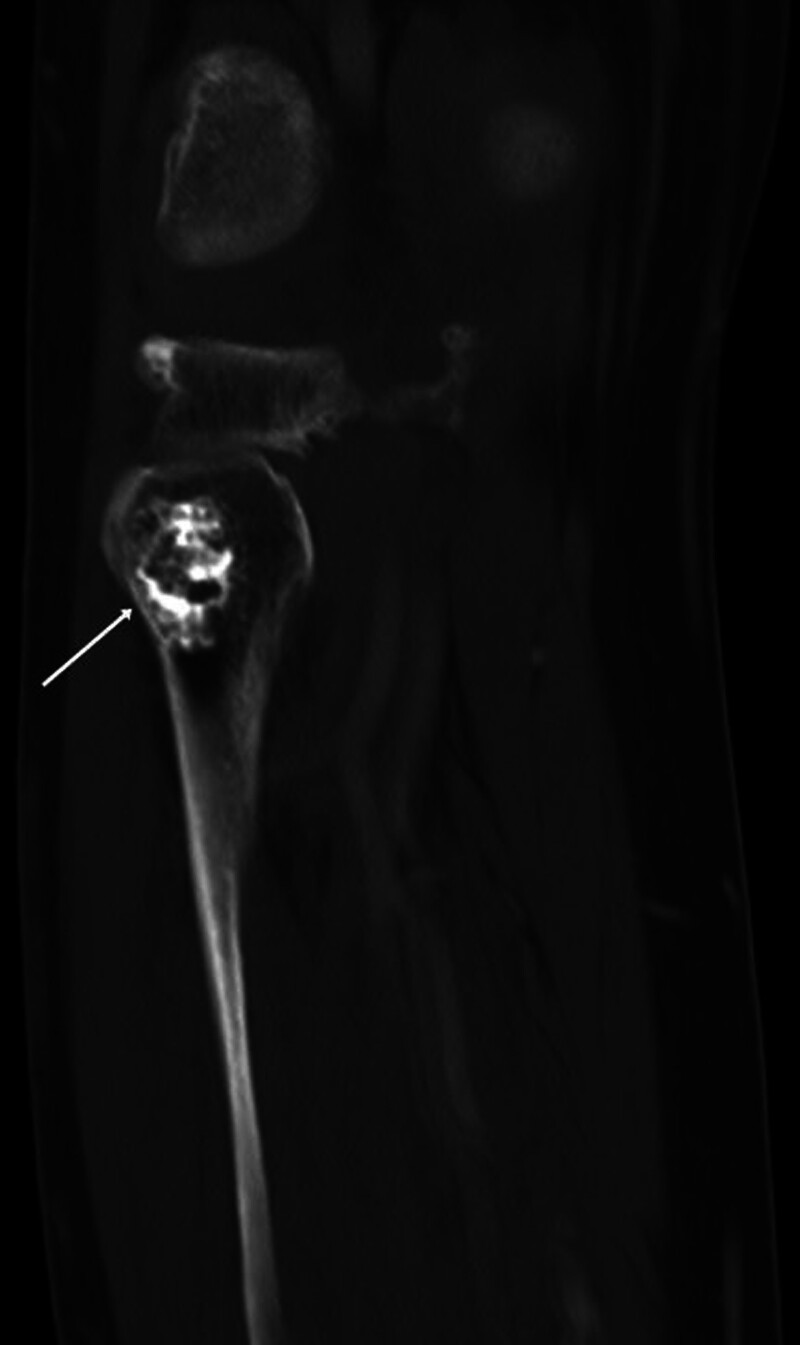
An intramedullary bone lesion containing calcifications in the proximal fibula compatible with enchondroma.

The current study was approved by the Local Ethics Committee (protocol number: 2024/03-53, date: February 13, 2024). Due to the retrospective nature of the study, informed consent was waived. The picture archiving and communication systems system and pathology database for the period between 2013 and 2024 were scanned for patients with enchondroma and chondrosarcoma. The inclusion criteria (Fig. 1) were as follows: pathologically proven low-grade chondrosarcoma (n = 70) (Fig. 2); pathologically proven enchondroma (n = 30); availability of a preoperative CT image (n = 44); patients with typical CT findings of enchondroma, such as sharply defined margins, chondroid calcifications, and unchanged size in at least a 3-year follow-up (n = 15) (Fig. 3). CT images with artifact (n = 3) and patients without CT imaging (n = 56) were excluded from the study.

Every CT scan was performed using a GE OPTIMA 128-slice CT scanner. The following were the CT scan parameters: 0.625 mm slice thickness, 25 to 40 cm field of view, 250 to 300 mAs, and 120 kV voltage.

### 2.1. Radiomic feature extraction

**Figure 4.**
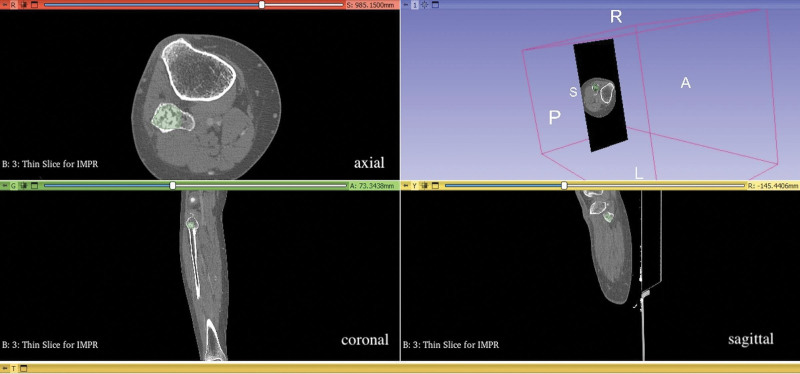
Manuel segmentation of enchondroma in fibula with 3D slicer program.

Axial thin slice noncontrast CT images (0.625 mm slice thickness) were uploaded to the 3D Slicer 4.10.2 program (www.slicer.org) with a “DICOM (Digital Imaging and Communications in Medicine)” format.^[14]^ Coronal and sagittal planes were obtained with multiplanar reformation. The uploaded images were standardized using ±3 sigma normalization and an N4ITK bias field correction filter. Tumor volume segmentation was carried out independently and manually using the 3D Slicer 4.10.2 software by 2 musculoskeletal radiologists, M.Y. and H.Y., with 7 and 13 years of experience, respectively. The tumor area was segmented along its entire volume in the axial, coronal, and sagittal planes (Fig. 4). The radiomic features were extracted by the Radiomics module of the 3D Slicer software. Using the Radiomics application, 14 shape-based features, 18 first-order features, 14 gray-level dependence matrix features, 16 gray-level run length matrix features, 24 gray-level co-occurrence matrix-based features, 5 neighboring gray-tone difference matrix features, and 16 gray-level zone size matrix features were obtained. Eventually, a total of 107 features were obtained for each patient from the CT images. The collected features are categorized according to the published records of PyRadiomics (https://pyradiomics.readthedocs.io/en/latest/features).

### 2.2. Statistical analysis

The statistical analysis for each feature was conducted using IBM SPSS version 25.0 (IBM Corp., Armonk, NY). The age and gender parameters of patients with chondrosarcoma and endochondroma were compared. A *P* value < .05 was considered statistically significant. To assess interobserver reliability, the intraclass correlation coefficient (ICC) was applied to estimate absolute agreement between the 2 radiologists. Good (ICC = 0.75–0.9) and excellent (ICC ≥ 0.9) interobserver reliability^[15]^ were obtained for the 107 features from each patient in the current study. Therefore, the machine learning classification was carried out with all 107 features.

### 2.3. Machine learning analysis

Support vector machine, naive Bayes, random forest, k-nearest neighbor (Knn), logistic regression, tree, and neural network algorithms in the ‘Orange Data Mining’ program version 3.24 (https://orange.biolab.si/) were applied for classification using machine learning.^[16]^ As an internal validation technique, a stratified 10-fold cross-validation was used. As a feature reduction method, the algorithm-based information gain method was used and classification was performed by selecting the 5 most important features to separate the 2 groups. Area under the curve (AUC), F1 score, precision, classification accuracy (CA), specificity, and recall values were used to detect the performance of the models. For each model, receiver operating characteristic (ROC) curves were generated.

## 3. Results

Thirty patients with enchondroma (17 female, 13 male) and 26 patients with low-grade chondrosarcoma (15 female, 11 male) were included in the study. The age range of patients with enchondroma was 20 to 72 years (44.90 ± 12.61). The age range of patients with low-grade chondrosarcoma was 25 to 77 years (47.19 ± 11.67). Regarding age and gender, no significant difference was detected between the 2 groups (*P* > .05).

**Figure 5.**
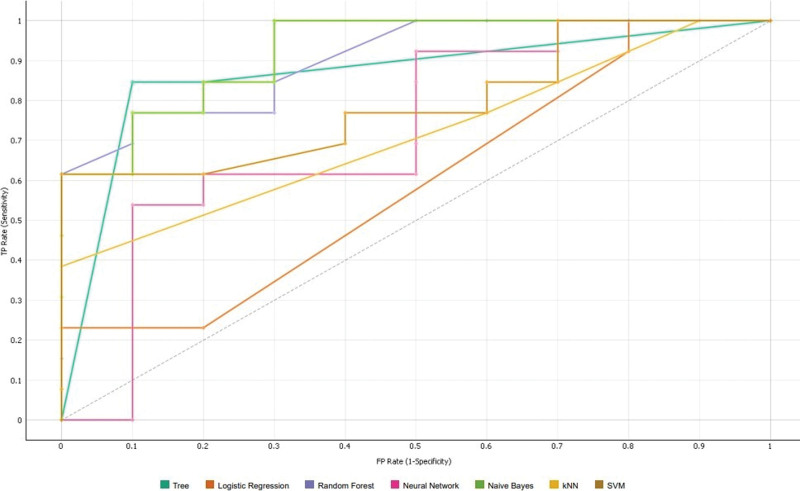
ROC curves of 7 models using all features. ROC = receiver operating characteristic.

Of the 30 enchondromas, 15 were located in the femur, 9 in the humerus, and 6 in the fibula. Of the 26 low-grade chondrosarcomas, 11 were located in the pelvis bones, 9 were in the femur, 3 were in the humerus, and 3 were in the spine. A total of 107 features were extracted from each patient’s 3D CT image. The AUC, precision, F1 score, CA, recall, and specificity values were determined with the datasets of both radiologists using 7 different machine learning classifications (Tables 1 and 2). ROC curves for all models were generated with the first radiologist’s dataset (Fig. 5). Classification using all features showed that naive Bayes was the best model for discriminating between chondrosarcoma and enchondroma when the evaluation was based on AUC values (AUC = 0.950). The best recall and specificity values were obtained with the tree model (0.846 and 0.900, respectively). A confusion matrix for the tree model was also created (Table 3).

**Table 1:** Performance of the machine learning models to distinguish between low-grade chondrosarcoma from enchondroma when all features were used (first radiologist’s dataset).

Model	AUC	Precision	F1	CA	Recall	Specificity
Naive Bayes	0.950	0.909	0.833	0.826	0.769	0.900
Random Forest	0.925	0.769	0.769	0.739	0.769	0.700
Support vector machine	0.867	0.600	0.727	0.609	0.923	0.200
Tree	0.850	0.917	0.880	0.870	0.846	0.900
Knn	0.808	0.625	0.690	0.609	0.769	0.400
Neural network	0.800	0.706	0.800	0.739	0.923	0.500
Logistic regression	0.650	0.600	0.333	0.478	0.231	0.800

AUC = area under curve, CA = classification accuracy, Knn = k-nearest neighbor.

**Table 2:** Performance of the machine learning models to distinguish between low-grade chondrosarcoma from enchondroma when all features were used (second radiologist’s dataset).

Model	AUC	Precision	F1	CA	Recall	Specificity
Naive Bayes	0.950	0.909	0.833	0.826	0.769	0.900
Tree	0.850	0.917	0.880	0.870	0.846	0.900
Support vector machine	0.867	0.600	0.727	0.609	0.923	0.200
Random Forest	0.858	0.786	0.815	0.783	0.846	0.700
Knn	0.808	0.625	0.690	0.609	0.769	0.400
Neural network	0.800	0.706	0.800	0.739	0.923	0.500
Logistic regression	0.650	0.600	0.333	0.478	0.231	0.800

AUC = area under curve, CA = classification accuracy, Knn = k-nearest neighbor.

**Table 3:** Confusion matrix of tree model using all features (first radiologist’s dataset).

Actual	Predicted		
	Low-grade chondrosarcoma	Enchondroma	Total
Low-grade chondrosarcoma	22	4	26
Enchondroma	3	27	30
Total	25	31	56

**Figure 6.**
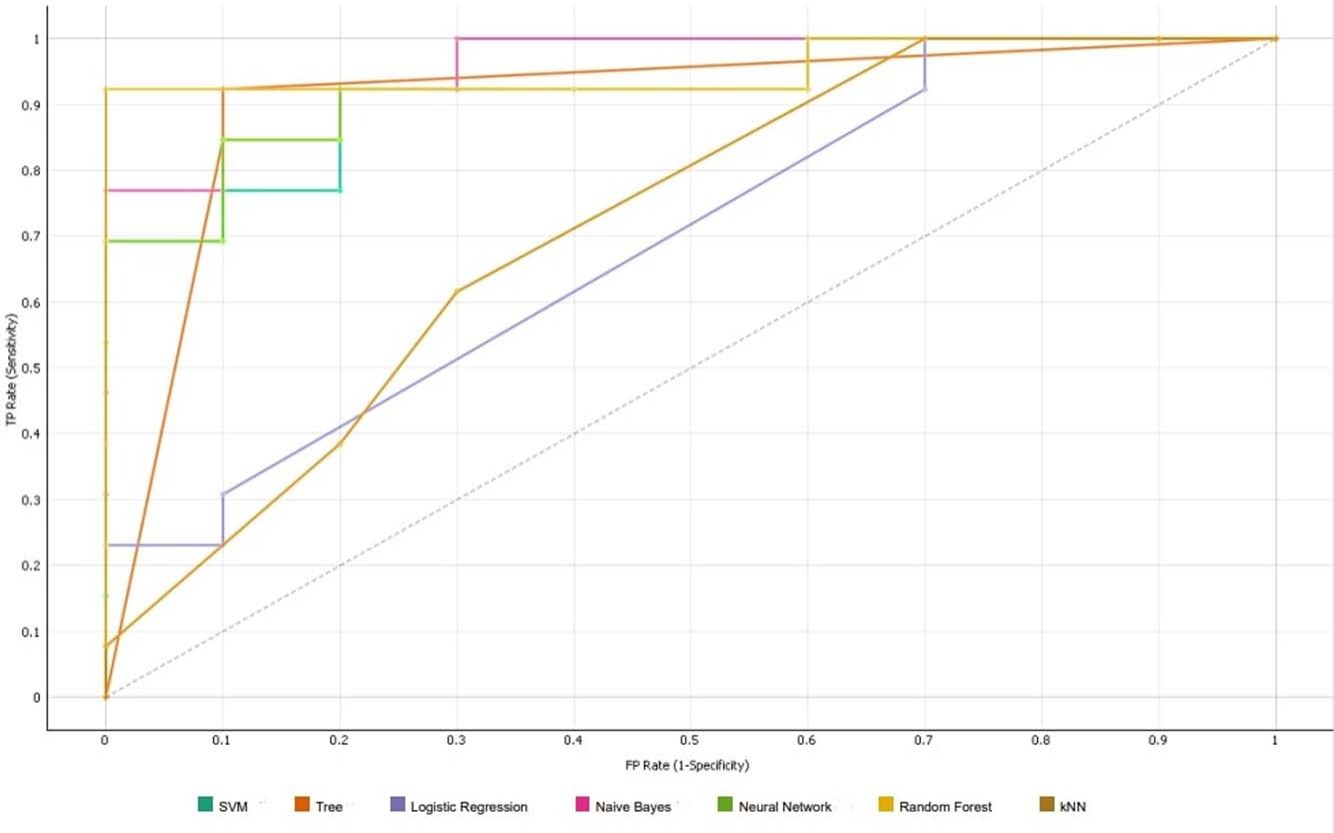
ROC curves of 7 models using 5 features. ROC = receiver operating characteristic.

In the first radiologist’s dataset, the algorithm-based information gain method indicated that the 5 most important features were coarseness (neighboring gray-tone difference matrix), 10th percentile (first-order feature), energy (first-order feature), total energy (first-order feature), and sphericity (shape-based feature). The AUC, precision, F1 score, CA, recall, and specificity values were determined for the 7 models using these 5 features (Table 4), and the ROC curve of all the models was created (Fig. 6). Classification using the 5 features showed that random forest was the best model for discriminating between chondrosarcoma and enchondroma when evaluated based on AUC values (AUC = 0.967). The best recall and specificity values were obtained with the random forest model (0.923 and 0.900, respectively). The confusion matrix of the random forest model was created (Table 5).

**Table 4:** Performance of the machine learning models when 5 features were used to distinguish between low-grade chondrosarcoma from enchondroma (sorted according to AUC values).

Model	AUC	Precision	F1	CA	Recall	Specificity
Random Forest	0.967	0.923	0.923	0.913	0.923	0.900
Naive Bayes	0.950	0.909	0.833	0.826	0.769	0.900
Neural network	0.917	0.917	0.880	0.870	0.846	0.900
Support vector machine	0.900	0.909	0.833	0.826	0.769	0.900
Tree	0.900	0.917	0.880	0.870	0.846	0.900
Knn	0.783	0.727	0.667	0.652	0.615	0.700
Logistic regression	0.650	0.800	0.444	0.565	0.308	0.900

AUC = area under curve, CA = classification accuracy, Knn = k-nearest neighbor.

**Table 5:** Confusion matrix of random forest model using 5 selected features.

Actual	Predicted		
	Low-grade chondrosarcoma	Enchondroma	Total
Low-grade chondrosarcoma	24	2	26
Enchondroma	3	27	30
Total	27	29	56

## 4. Discussion

The purpose of this study was to evaluate the effectiveness of CT-based radiomic and machine learning models in discriminating between enchondroma and low-grade chondrosarcoma. In our study, classification using the 5 most important features showed that random forest was the best model for discriminating between chondrosarcoma and enchondroma. An AUC value of 0.967, a recall of 0.923, and a specificity of 0.900 were obtained with this model.

Chondrosarcoma is most often observed in older people^[4]^ and is usually treated with either curettage or resection. Surgery is generally not required for enchondromas.^[17]^ Therefore, it is important to distinguish enchondroma from chondrosarcoma.

MRI is frequently utilized to identify and distinguish chondrosarcoma from enchondroma; however, its diagnostic capacity is restricted.^[8]^ In addition, due to the similar pathological features of enchondroma and chondrosarcoma, histopathological distinction between the 2 tumors can be challenging even among experienced musculoskeletal pathologists.^[8]^

Recently, radiomics has become a popular tool for the diagnosis of disease and for staging different types of cancer.^[18]^ Some studies have employed radiomic analysis based on the MRI characteristics of cartilaginous bone lesions. MRI-based texture analysis was utilized by Lisson et al to distinguish between enchondromas and low-grade chondrosarcomas.^[10]^ Pan et al used 3 radiomic analyses based on T1-weighted image (WI), fat-suppressed T2-WI, and T1-WI + T2-WI sequences to differentiate between chondrosarcoma and enchondroma.^[19]^ Gitto et al used MRI radiomics-based machine learning to differentiate grade II chondrosarcomas from atypical cartilaginous tumors.^[20]^ Erdem et al used radiomic features of T1-WI and proton density MRI to differentiate enchondroma from chondrosarcoma.^[11]^ These studies are all MRI-based and achieved high accuracy rates. Additionally, Yoon et al^[12]^ used SPECT/CT radiomics analysis to differentiate between low-grade chondrosarcoma and enchondroma while Gitto et al used CT radiomics and machine learning to classify atypical cartilaginous tumors from higher-grade chondrosarcomas.^[21]^

To our knowledge, the present study is the first to use 3D CT-based radiomics and machine learning to distinguish low-grade chondrosarcoma from enchondroma. The AUC value of 0.950 was obtained with the naive Bayes model using all features. A specificity of 0.900 and a recall value of 0.846 were obtained with the tree model using all features. With feature reduction, AUC values of ≥0.900 were obtained in 5 different models (naive Bayes, neural network, support vector machine, tree, and random forest) and the highest AUC was obtained with the random forest model. After feature reduction, a recall value of 0.923 and a specificity value of 0.900 were obtained with the random forest model. Additionally, the ICC indicated the presence of good to excellent interobserver reliability for 107 radiomic features of each image.

Lisson et al used MRI-based texture analysis to distinguish enchondroma from low-grade chondrosarcoma and indicated a significant difference in 4 texture parameters in different MRI sequences. These authors reported that the kurtosis parameter in contrast-enhanced T1WI had the strongest capability for discrimination, with an AUC value of 0.876.^[10]^ Yoon et al reported that zone-length nonuniformity (ZLNU, AUC = 0.090) and coarseness were the 2 most important radiomic features in discriminating between low-grade chondrosarcoma and enchondroma using SPECT/CT radiomics analysis.^[12]^ The highest AUC value obtained in the current study was 0.967, indicating further improvement.

The small sample size and the retrospective design of the study are the limitations of our study. Additionally, all images were collected from a single hospital. In the future, a multicenter investigation with a larger sample size should be carried out.

In conclusion, a CT-based radiomics analysis was successfully used to distinguish between low-grade chondrosarcoma and enchondroma in the current study, and the best-performing machine learning model was determined. Machine learning models based on CT radiomics can be used to differentiate between low-grade chondrosarcoma and enchondroma.

## Author contributions

**Conceptualization:** Mustafa Yildirim, Hanefi Yildirim

**Data curation:** Mustafa Yildirim, Hanefi Yildirim

**Investigation:** Mustafa Yildirim

**Methodology:** Mustafa Yildirim

**Software:** Mustafa Yildirim

**Writing – original draft:** Mustafa Yildirim

**Supervision:** Hanefi Yildirim
